# Combined conceptual and perceptual control of visual attention in search for real-world objects

**DOI:** 10.3758/s13414-025-03116-4

**Published:** 2025-09-25

**Authors:** Brett Bahle, Kurt Winsler, John E. Kiat, Steven J. Luck

**Affiliations:** Department of Psychology and Center for Mind & Brain, University of California, Davis, 267 Cousteau Place, Davis, CA 95618 USA

**Keywords:** Visual search, Template, Perceptual similarity, Conceptual similarity, Cognitive and attentional control

## Abstract

When we search for an object in the natural visual environment, we sometimes know exactly what the object looks like. At other times, however, we know only the category of the object. For example, if we are looking for our own bath towel, we might know that it is brown and is folded into a rectangle. However, if we are looking for a towel in a friend’s house, we might not know its color or whether it is folded or lying in a clump. Consequently, we may sometimes be able to use specific perceptual features to guide search, but some search tasks are so conceptual in nature that the relevant visual features are difficult to specify. Here, we found that eye-movement patterns during visual search could be predicted by perceptual dimensions derived from crowd-sourced data (THINGS), but only when observers had previously seen the specific target object. When only the category of the desired object was known (because the observer had never seen the specific target), eye-movement patterns were predicted by conceptual dimensions derived from a natural language processing model (ConceptNet), and perceptual features had no significant predictive ability once the conceptual information was statistically controlled. In addition, as observers gained experience searching for a specific exemplar of a category, they became progressively more reliant on perceptual features and less reliant on conceptual features. Together, these findings provide novel evidence that conceptual information can influence search, especially when the precise perceptual features of an object are unknown.

**Supplementary Information:**

The online version contains supplementary material available at 10.3758/s13414-025-03116-4.

## Introduction

Visual search is important to understand because it is both a natural, ubiquitous activity (e.g., searching for a banana in a grocery store) and a specialized task involving potentially life-changing outcomes (e.g., a radiologist searching for a tumor in a medical scan). It has long been studied using simplified stimuli like T targets among L distractors (e.g., Treisman & Gelade, 1980). Such research has been enormously successful, leading to influential cognitive models, like Guided Search (Wolfe, 1994, 2021), whose predictions closely match human data under a wide variety of conditions. Importantly, in this model, search is guided toward relevant locations in a search scene based solely on the perceptual features available in the display.

For example, in a search for a red square among other shapes colored blue or red, one might hold a “guiding template” in visual working memory that contains the features “red” and “square.” Indeed, many descriptions of attentional guidance begin with an example such as “Imagine you are searching for your friend in a crowd, and you know your friend is wearing a blue hat” or “Imagine you are shopping for bananas, so you restrict your attention to yellow items.” Once a search display appears, attention is preferentially directed toward locations in the visual field to the extent that they match these guiding features. Attention may also be attracted by perceptual features associated with motivationally relevant outcomes, such as rewards (e.g., Anderson et al., 2011). Even in these cases, the guiding features are restricted to the relatively low-level perceptual features associated with the motivational outcomes.

Once a location is attended, a target-decision operation occurs in which a “target template” is compared with the object at the current location to determine whether this attended item is indeed the target of search. Although this later, target-decision operation is not as well modeled as the guidance operation of search, it is typically thought to be driven largely by low-level perceptual features (see, e.g., Yu et al., 2022a).

More recent work has explored search for real-world objects. This type of search raises the question of whether search may be guided by the semantic or conceptual properties of objects in addition to their perceptual features.^1^ Humans learn a rich network of concepts over the course of life that are organized by their meaning (Collins & Loftus, 1975). Moreover, complex visual features can be processed to some extent in peripheral vision, outside the focus of attention (Braun & Sagi, 1990; Lee et al., 1997). Thus, even relatively simple feedforward processing of visual information from visual cortex to higher-level areas may contain sufficient information to identify objects in the periphery and activate their conceptual representations. Note that, even without an attentional mechanism, purely feedforward convolutional neural networks designed to mimic the architecture of the human brain can extract object identity in this way (Krizhevsky et al., 2012). When a conceptual representation of an object is activated in this manner, the attentional priority of the location containing that object could be increased if the conceptual representation of the object is related to the target category. In this way, conceptual information about objects could be activated preattentively and then used to guide shifts of attention. This makes it possible to envision how attentional guidance could work for tasks such as “Imagine you are shopping to furnish a dorm room” in which the searcher would restrict attention to desks, chairs, rugs, posters, and other items that might be found in a dorm room.

Some researchers have argued that such higher-level representations do not guide attention during search (Vickery et al., 2005; Wolfe et al., 2011; Wolfe & Horowitz, 2017; Wolfe, 1994, 2021). For example, a classic diagnostic for whether a feature can guide search is whether it “pops out” when that feature is unique in a search display, but Wolfe et al. (2011) found that searching for an object category (e.g., “lollipop”) does not show the characteristic “flat” search slope (RT/set size) corresponding to pop-out search. However, a lack of popout does not mean a lack of guidance; it can simply mean that the target-distractor similarity is high enough that guidance is imperfect (see Duncan & Humphreys, 1989; Palmer, 1995). Another argument against conceptual guidance of attention is that some evidence suggests that focal attention may be required to determine an object’s meaning (Lachter et al., 2004). For example, using a spatial priming paradigm, Lachter et al. (2004) demonstrated that stimuli produced a lexical priming effect only when they appeared in a focally attended location, with little-to-no priming outside this location. However, these results are based on comparing processing for an object presented inside versus outside of a narrowly focused area, whereas search tasks tend to begin with attention broadly distributed across the display. When attention is broadly distributed at the onset of a scene, other evidence indicates that high-level category information can be extracted rapidly and in parallel (Rousselet et al., 2002; Vogel et al., 2005). This establishes the plausibility of the hypothesis that conceptual information can be extracted preattentively and used to guide attention.

Several studies have attempted to provided evidence that conceptual or semantic information does, in fact, guide attention during search (Belke et al., 2008; Cimminella et al., 2020; de Groot et al., 2016; Moores et al., 2003; Nako et al., 2014; Nuthmann et al., 2019; Yeh & Peelen, 2022). These types of studies typically find that attention is more likely to be directed toward an object that is visually dissimilar but semantically related to the target of search (e.g., fixating a helmet when searching for a bike) than to a visually dissimilar but semantically unrelated object (e.g., a hammer). However, these previous studies suffer from some important limitations. In some studies, for example, participants saw the same specific target and nontarget stimuli many times, creating the possibility that they learned the visual features of the objects and used those features (rather than the conceptual information) to guide search (Belke et al., 2008; Cimminella et al., 2020; Moores et al., 2003; Nako et al., 2014; Yeh & Peelen, 2022). In addition, many studies have relied on qualitative, intuitive definitions of conceptual and perceptual relatedness rather than rigorously quantifying the perceptual and conceptual relatedness of the target and the distractors. Other studies quantified perceptual and conceptual similarity, but they still treated a given nontarget object as being *either* conceptually *or* perceptually similar to the target (de Groot et al., 2016; Nuthmann et al., 2019).

Another important issue is that objects that are conceptually related also tend to be perceptually related. For example, classic research by Rosch et al. (1976) demonstrated that objects in the same category possess similar shapes. Consequently, distractors treated as being conceptually similar to the target in the previous studies were also likely to be more perceptually similar to the target than the “unrelated” distractors. Thus, the finding that the conceptually related targets attracted attention more than the unrelated distractors might have been driven by perceptual similarity rather than conceptual relatedness. Accordingly, although prior research has made progress toward establishing that conceptual information can be used to guide visual search, the evidence is not yet conclusive. Most notably, it is important to examine the allocation of attention the first time a participant is asked to search for an item of a given category in a given task, before they have seen the actual target exemplar, and it is important to ask whether the conceptual similarity of a distractor to a target impacts the allocation of attention after carefully controlling for perceptual similarity.

Here, we developed a new approach to testing whether conceptual information can influence the guidance of overt attention^2^ during visual search that was designed to overcome the limitations of previous research. Specifically, we developed search tasks in which we could directly compare performance when participants:

1. Had precise perceptual information regarding the particular object category they were searching for by cuing search with an exact picture-cue of the target, or
2. Had no precise perceptual information regarding the target by cuing them with a category-cue (e.g., “grasshopper”).

We also looked at search performance separately for the first search for a category with a particular exemplar and for the 12th search for that same category and corresponding exemplar. Although even the category cue on the first search for a category restricted the available space of perceptual features the target could plausibly contain (grasshoppers are not ordinarily blue), such a situation minimized the extent to which perceptual information was available for search.

**Fig. 1:**
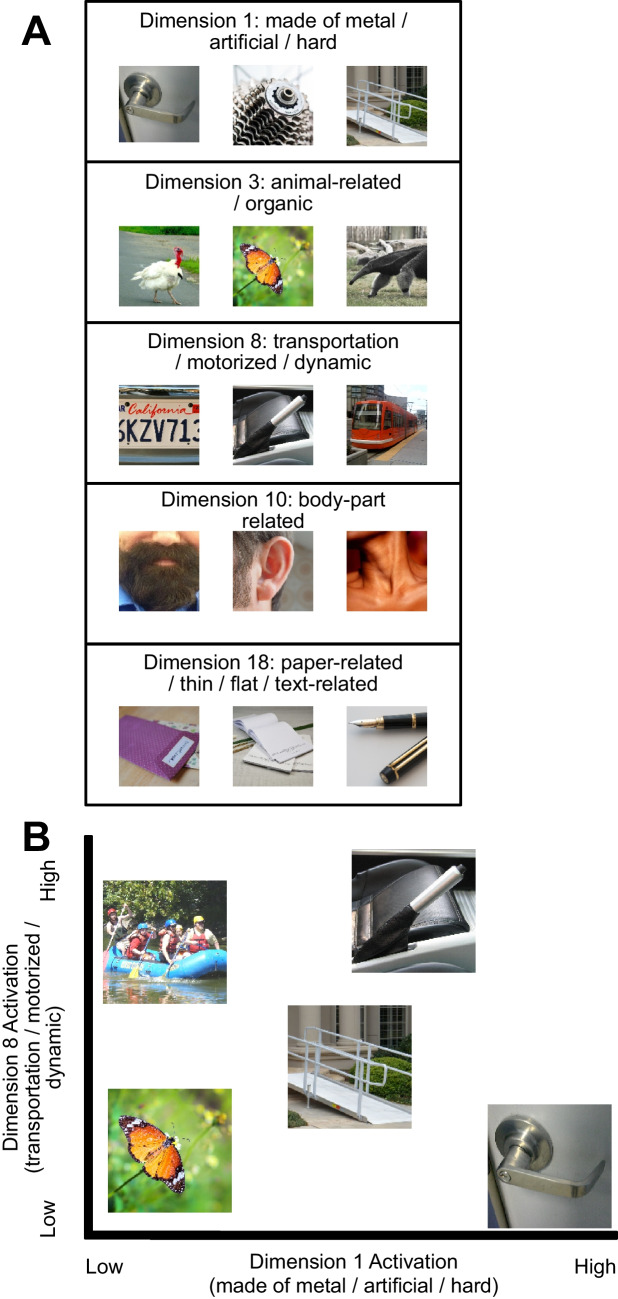
**A**) Stimuli from the present experiments whose activation is maximal on a given dimension. For example, in the Box labeled"Dimension 1: made of metal/artificial/hard", each image had its maximal activation on dimension 1 (among the 49 THINGS dimension). **B**) Example of how stimuli vary in their activation across dimensions. Here, we show five image of varying activtion on Dimensions 1 and 8 (drawn to scale) the butterfly has low activation on both Dimension 1 and Dimension 8; the raft has low activation on Dimension 1 but high activation on Dimension 8: the door-handle has high activation on Dimension 1 but low activation on Dimension 8: the handbrake has high activation on Dimension 1 and high activation on Dimension 8: the ramp has medium activation on both Dimension 1 and Dimension 8

Further, our approach allowed us to simultaneously quantify both the *continuous* perceptual similarity and the *continuous* conceptual similarity between the target and distractors. That is, a given target and the associated distractor(s) were simultaneously scored on both their perceptual and conceptual relatedness. To quantify perceptual similarity, we used a method developed by Hebart et al. (2020) that represents a given object (from a set of ~1,800 real-world objects) as a location in a 49-dimensional *embedding* space. The embedding space was created empirically using millions of human categorization judgments. Importantly, the dimensions derived through the training of the embedding space ranged from relatively low-level perceptual features (e.g., “red,” “round”) to more abstract perceptual features (e.g., “hard,” “body-part related”). This broad range of perceptual features mirrors the fact that feedforward perceptual processing in the human brain activates neurons with a broad range of feature sensitivities, ranging from simple features in V1 to complex features in inferotemporal cortex, all of which could potentially be used to control shifts of attention. The targets and distractors in our visual search experiments were drawn from this set of ~1,800 objects, allowing us to quantify the similarity between a target and a distractor as the nearness of the two object representations in the embedding space. The larger the Euclidean distance between the objects, the more perceptually dissimilar they are (see Fig. 1B for examples).

To quantify conceptual relatedness, we used *ConceptNet*, which is a semantic network trained on huge corpuses of text (including the entirely of the English contents of Wikipedia and Wiktionary). ConceptNet assigns a given word to a location in a 300-dimensional semantic embedding space (Speer et al., 2017). To use ConceptNet, we assigned a label (derived from the THINGS database from which the search images were used) to each target and distractor object, and we computed the Euclidean distance between a given target label and each distractor label in the ConceptNet space. This quantified the conceptual dissimilarity between the target and the distractor. We chose ConceptNet to quantify conceptual relatedness because, across several scoring metrics, the representations in this space map closely onto human semantic similarity judgments. For example, the distances between pairs of labels within the ConceptNet embedding space are highly correlated with human similarity judgments for those same pairs, with an *r* = 0.86 (Speer et al., 2017). These embedding values also score at state-of-the-art performance on various tasks that require understanding conceptual relatedness, such as determining which labels best complete an analogy (such as fire : hot :: ice : X) or choosing which of two sentences best constitute a sensible ending to a four sentence story (Speer et al., 2017).

Using the THINGS space and the ConceptNet space, we separately quantified both the perceptual similarity and the conceptual similarity of each target-distractor pair. By using both the perceptual and conceptual similarity values simultaneously in a multilevel statistical model to predict search performance, we could assess the extent to which each of them accounted for unique variance in behavioral performance. This made it possible to assess whether conceptual relatedness can predict search behavior after controlling for perceptual relatedness.

To preview the results, we found that search behavior was strongly predicted by conceptual relatedness when the searchers did not know the precise perceptual features of the search target, with little or no influence of perceptual relatedness. This role of conceptual information was observed both for the *attention-getting* ability of an object (i.e., the guidance stage of search) and the *attention-holding* ability of an object (i.e., the target-decision stage of search). In contrast, when the searchers already knew the precise perceptual features of a target, search behavior was strongly predicted by perceptual relatedness, with little or no influence of conceptual relatedness. Our work provides compelling new evidence that visual search can be driven by conceptual information under conditions that reflect many real-world searches, in which the searcher knows the conceptual category of a target but not its specific perceptual features.

## Experiment 1: The effect of conceptual and perceptual dissimilarity on saccade choice behavior

**Fig. 2:**
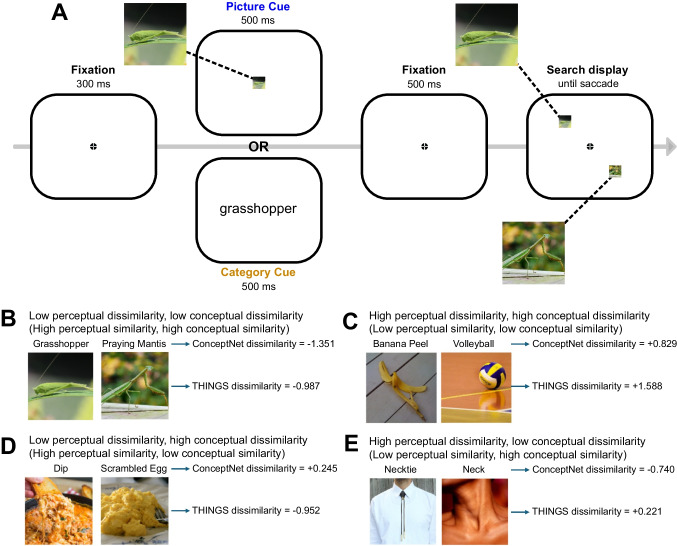
**A**) Sequence of events in Experiment 1. Stimuli are draw to scale. One group of participants saw picture cues and another group saw category cues. **B-E**) Example of target-distractor pairs with varying levels of perceptual and conceptual dissimilarity (with z-scored dissimilarity values)

In Experiment 1, we were primarily interested in assessing the extent to which the guidance of attention is influenced by conceptual and perceptual information. To do so, we had participants perform the two-item search task illustrated in Fig. 2A. Each trial began with a target cue that was either a picture cue (that exactly matched how the target appeared in the following display) or a category cue (a basic-category label for the target object). Such cues are well established as effective in guiding search (see, e.g., Maxfield & Zelinsky, 2012; Yang & Zelinsky, 2009). One group of participants received picture cues, and another received category cues.

Each search display contained one target and one distractor. Participants were instructed to make a speeded saccade to the target object, with the restriction that they could make only one saccade per display (because the trial ended shortly after gaze left central fixation). There were 80 target/distractor pairs that varied systematically in the conceptual and perceptual similarity of the paired items (see Fig. 2B-E). Each target was searched for 12 times throughout the course of the experiment (with a given target always appearing with the same distractor).

Our primary analysis examined the ability of the perceptual and conceptual similarity between the target and distractor to uniquely predict saccade choice accuracy, asking whether conceptual similarity predicted performance after controlling for perceptual similarity and whether perceptual similarity predicted performance after controlling for conceptual similarity. The trials were also coded in terms of cue type (picture vs. category cue) and exposure number (first vs. last trial with a given target-distractor pair). We expected that, if conceptual information can guide search, the probability of correctly shifting overt attention to the target would be greater when the target and distractor were conceptually dissimilar than when they were conceptually similar, even after controlling for the perceptual similarity between them. However, we expected that ability of conceptual similarity to predict performance would be present primarily when the searchers did not know the specific perceptual features of the target (i.e., the first search for a given target when cued with a category label). By contrast, when the searchers had seen the target, we expected that the probability of correctly fixating the target would be greater when the target and distractor were perceptually dissimilar than when they were perceptually similar, even after controlling for the conceptual similarity between them.

### Materials and methods

#### Participants

In all experiments, participants were recruited from the University of California-Davis community and received monetary compensation ($15/h) for their participation. All participants reported normal or corrected-to-normal visual acuity, were screened for color blindness, had no prior history of neurological conditions, and provided informed consent. All human subjects procedures were approved by the University of California-Davis Institutional Review Board.

We tested two groups of participants, one with picture cues and one with category cues. To ensure that the experiments were sufficiently powered, we ran a pilot experiment (N = 16; ten women, six men) that was identical to the picture-cue condition of Experiment 1. A power analysis on this dataset using G-Power indicated that a sample size of 16 would be sufficient to have.9 power for detecting the presence of an above-chance correlation between search accuracy and the THINGS-based similarity score. To be conservative, we ran 24 participants in each cuing condition. Two participants were replaced in the category-cue condition of Experiment 1 for having mean saccade accuracy less than an a priori rejection threshold of 60%; one participant was replaced in the category-cue condition for having mean saccade latency greater than an a priori rejection threshold of 500 ms. The final gender breakdown of participants for the picture-cue condition was 16 women and eight men and for the category-cue condition was 19 women and five men.

#### Apparatus

Stimuli were presented at 100 Hz on an LCD monitor (resolution: 1,920 × 1,080 pixels; model: Dell U2412M; viewing distance: 100 cm) with a white background (X.X cd/m^2^). An empirically optimized black fixation point (Thaler et al., 2013) subtending 0.5 × 0.5° of visual angle was presented in the center of the screen. The experiment was controlled by MATLAB (MathWorks, Natick, MA, USA) using PsychToolbox (Brainard, 1997; Pelli, 1997) on a computer running Linux. Each participant’s right eye was monitored at 500 Hz using an SR Research Eyelink 1000+ eyetracker with a nine-point calibration.

#### Stimuli

Stimuli were selected from the set of 1,854 images used in Hebart et al. (2020), which were themselves drawn from the THINGS database (Hebart et al., 2019). In Experiment 1, the same 80 target-distractor pairs were used in both the category-cue and picture-cue conditions. We selected these pairs to obtain a broad distribution of perceptual similarity (which also led to a broad distribution of conceptual similarity). Specifically, we first computed the Euclidean distance in the 49-dimensional THINGS space between all possible pairs of the 1,854 images (i.e., the dissimilarity between the pairs). We then sorted all pairs by distance and randomly selected 20 pairs at four different levels of dissimilarity (0.4, 0.8, 1.6, and 3.2 in the arbitrary units of the embedding space). We could not find pairs with these exact levels of dissimilarity, so we algorithmically found the pairs that were closest to these dissimilarity levels. For example, for the 0.4 level, we first tried to find pairs that were within 0.05 of the target level (i.e., 0.35–0.45), then 0.10 of the target level, and so on. In addition, we did not allow any individual item to occur in more than one pair.

As illustrated in Fig. 2A, the cue in the picture-cue condition was the picture of the search target for that trial (subtending 3.1° × 3.1°). In the category-cue condition, the cue was a basic-level category label for the search target, rendered in a black Arial font subtending 1.1–2.3 degrees. The category labels were taken from the labels provided by the THINGS database unless the labels were ambiguous, in which case we used a more descriptive term. For example, in the THINGS database, a picture of a baby cow is listed as “calf,” which could be interpreted (correctly) as a baby cow or (incorrectly) as a body part, so we used an unambiguous label (e.g., “baby cow”). The full list of labels is provided in Online Supplementary Material (OSM) Table S4 (sorted by THINGS dissimilarity) and Table S5 (sorted by ConceptNet dissimilarity). Cues were presented in the center of the display.

The search display consisted of the central fixation dot and the two search images (target, distractor). The target and distractor were presented opposite each other on a virtual circle that was centered at fixation, with a radius of 3.875°. The radial location of the pair of objects on the circle was randomly selected on each trial.

### Procedure

Each trial followed the sequence depicted in Fig. 2: the central fixation dot was presented for 300 ms, followed by a 500-ms cue, then a 500-ms central fixation dot, and then the search display. Participants were instructed to maintain central fixation until they identified the target and then to make a saccade towards it. The search display and the fixation dot terminated 200 ms after gaze left central fixation. This provided sufficient time for eye movement completion and verification of the identity of the fixated item, but it did not provide time for a second saccade. Thus, participants made a single saccade on each trial, either toward the target or toward the distractor. Incorrect saccades triggered an “Incorrect!” message for 5 s in red font. The relatively long duration of this message on error trials was intended to incentivize correct eye movements. Correct responses simply ended the trial. The next trial began after a 400-ms inter-trial interval.

Each pair of objects was searched 12 times, for 960 total trials, split into six blocks of 160 trials. The order of the 960 trials was randomized separately for each participant.

### Data analysis in Experiment 1

Saccades were scored as either directed toward the target or the distractor in the following way. First, we monitored for the first eye position sample that was outside a 2° virtual circle centered at central fixation. Once found, the distance between that sample position and the target/distractor were computed, with the smaller distance being the object the saccade was directed to. All trials were included in the saccade choice accuracy analyses. For the latency analyses, 20.9% of trials were removed because (1) an incorrect saccade choice was executed or (2) the latency was greater than 2.5 SDs away from the participant’s mean. We describe the accuracy results in terms of percent correct, but the statistical analysis was actually performed on single-trial data (incorrect coded as 0; correct coded as 1) using a logit transformation.

The saccade choice accuracy data were analyzed using a logistic mixed effects (LME) regression model in R using the lme4 package (Bates et al., 2015; see https://osf.io/su9nf/ for the R code). We asked whether the choice on each trial could be predicted by the dissimilarity (distance) between the target and distractor in both the THINGS perceptual space and the ConceptNet conceptual space, as described above. For both spaces, dissimilarity was quantified as the Euclidean distance between the vectors for the target and distractor labels in the multi-dimensional embedding space. The distances were then z-scored (separately within each space). In addition to THINGS Dissimilarity and ConceptNet Dissimilarity, the model included fixed effects for Exposure Number and Cuing Condition. The model also contained the interactions between all fixed effects. As is justified in the next section, only the first and last exposures were included in this LME analysis (Exposure Numbers 1 and 12). Exposure Number and Cuing Condition were dummy-coded with the first exposure, category-cue condition as the reference group. Significance was tested for the marginal effects of each variable using Wald *z*-tests. The random effect structure included random intercepts for participants and items (where item corresponds to each unique target/distractor pair), random participant and item slopes for the effect of Exposure Number, with an unstructured covariance matrix for all specified random effects.

## Results

### Overall choice accuracy across the 12 exposures

**Fig. 3:**
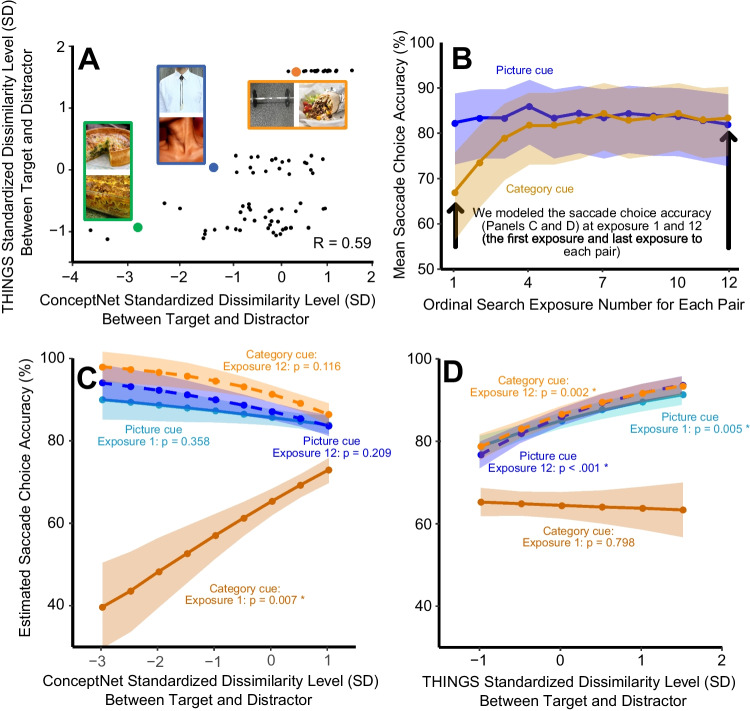
**A**) Scatter plot of the relationship between THINGS and ConceptNet standardized dissimilarity scores for the target-distractor pairs used in Experiment 1, inset with example pairs. **B**) Observed mean saccade choice accurancy over exposures to the target-distractor pairs, separately for the category-label-cue and the picture-cue. **C** and **D**) Estimated saccade choice accurancy from the statistical model, showing the effect of ConceptNet standardized dissimilarity (**C**) level and THINGS dissimilarity level (**D**) between target and distractor pairs on saccade choice accurancy. Note that both predictors were fit in the same model. Model fits are plotted separately for the category-label-cue and the picture-cue conditions for Exposure 1 (the first search for each pair) and Exposure 12 (the last search for each pair). Shaded regions show ±1 standard error

Figure 3B shows the observed mean saccade choice accuracy across the 12 exposures, averaged across stimulus pairs. For the first exposure, search performance was much better following a picture cue than following a category cue. However, by the fifth exposure (and all subsequent exposures), behavioral accuracy was nearly identical across the two cuing conditions. Our statistical analyses included only the first and 12th exposures to avoid making any assumptions about the shape of the learning curve. This choice was also optimal for testing our specific scientific hypotheses, because it allowed us to compare performance when participants had no direct knowledge of the visual properties of the target (Exposure 1 in the category-cue condition) with performance when they had substantial knowledge of the visual properties of the target (Exposure 12 in both cuing conditions). We have made the data from all 12 exposures available at https://osf.io/su9nf/ for anyone who is interested in modeling the learning process, and OSM Fig. S2 shows the results from separate analyses for each of the 12 exposures.

### Effects of conceptual and perceptual similarity on choice accuracy

The full statistical model output for the main LME analysis is provided in OSM Table S1. Here, we focus on the marginal effects transformed from logits into probabilities of saccade choice accuracy (although the LME was performed in logit space using the binary single-trial accuracy data). Figures 3C and D illustrate the model results for ConceptNet dissimilarity and THINGS dissimilarity, respectively (from a single model that included both variables).

#### Category-cue condition

For the first exposure in the category-cue condition, choice accuracy was strongly predicted by the conceptual dissimilarity between the target and distractor but not by the perceptual dissimilarity. Specifically, a 1-SD increase in ConceptNet dissimilarity predicted a 7.5% increase in saccade choice accuracy (*z* = 2.72, *p* =.007; Fig. 3C), whereas THINGS dissimilarity did not significantly predict choice accuracy for the first exposure in the category-cue condition (*z* = −0.26, *p* =.798; Fig. 3D). By the 12th exposure of a category cue, however, this pattern reversed: a 1-SD increase in THINGS dissimilarity predicted a 5.2% increase in saccade choice accuracy (*z* = 3.41, *p* <.001), whereas ConceptNet dissimilarity did not significantly predict performance (*z* = −1.26, *p* =.209). These saccade choice accuracy results were paralleled by modest variations in saccade latency across THINGS and ConceptNet dissimilarity levels (see below and OSM Fig. S1 and Table S2).

#### Picture-cue condition:

The results for both the first and the 12th exposure in the picture-cue condition were very similar to the results from the 12th exposure in the category-cue condition, with perceptual similarity strongly predicting accuracy and conceptual similarity not being a significant predictor. Specifically, THINGS dissimilarity significantly predicted choice accuracy in the picture-cue condition (first exposure: *z* = 2.81, *p* =.005; 12th exposure: *z* = 3.41, *p* <.001), but ConceptNet dissimilarity did not (first exposure: *z* = −0.92, *p* =.358; 12th exposure: *z* = −1.26, *p* =.209). Saccade latencies in the picture-cue condition were largely invariant across THINGS and ConceptNet dissimilarity levels (see OSM Fig. S1 and Table S1).

#### Interactions

The different patterns of slopes shown in Fig. 3C and D were supported by a significant interaction between ConceptNet dissimilarity and Cuing Condition (*z* = −3.37, *p* <.001) and between THINGS dissimilarity and Cuing Condition (*z* = 3.11, *p* =.002). Moreover, the three-way interaction between ConceptNet dissimilarity, Cuing Condition, and Exposure Number was also significant (*z* = 2.45, *p* =.014). The three-way interaction between THINGS dissimilarity, Cuing Condition and Exposure Number did not reach significance (*z* = −1.74, *p* =.081). Note that these patterns developed gradually across exposures (see OSM Fig. S2).

### Effects of conceptual and perceptual similarity on saccade latency

This experiment was designed to examine which item was chosen for the saccade on each trial, so we provide only a brief description of the latency results (limited to trials with correct choices). As shown in OSM Fig. S1, latencies were slower in the category-cue condition than in the picture-cue condition on the first exposure, but latencies in the category-cue condition became much faster by the 12th exposure. The latencies were largely unrelated to the conceptual and perceptual similarity, except for the 12th exposure in the category-cue condition, in which latencies were faster when the target and distractor were more perceptually dissimilar and slower when they were more conceptually similar. However, as shown in OSM Table S2, neither the conceptual dissimilarity nor the perceptual dissimilarity exhibited a significant three-way interaction with cuing condition and exposure number. Thus, as intended by our experimental design, perceptual and conceptual similarity mainly predicted the accuracy of the saccades rather than the latency.

## Discussion

We found that when searchers knew only the category of the search target, and had never seen the specific instance of the target (Exposure 1 of the category-cue condition), their search behavior was strongly predicted by the conceptual similarity between the target and distractor. That is, participants were reasonably accurate at directing their gaze toward the target when the target and distractor were conceptually dissimilar, but they were quite inaccurate when the target and distractor were conceptually related. Our analyses controlled for the degree of perceptual dissimilarity between the target and distractor, so this finding cannot easily be explained by the fact that perceptual and conceptual similarity were correlated (see Fig. 3A). These results provide new evidence that conceptual information can be used to guide attention during visual search, at least under some conditions.

One might object that the THINGS embedding space does not fully capture the perceptual information used to guide search, and that controlling for the perceptual similarity using this embedding space was therefore insufficient to fully control for perceptual similarity when assessing the effects of conceptual similarity. Although it is likely that our analyses did not completely control for *all* perceptual similarity effects, we find that it is implausible that any residual uncontrolled perceptual similarity effects were sufficiently robust to entirely account for the very large effect of ConceptNet similarity observed for Exposure 1 of the category-cue condition. That is, even after variance explainable by THINGS was accounted for, accuracy went from near chance when ConceptNet similarity was low to approximately 75% correct when ConceptNet similarity was maximal (see Fig. 3C). It would be very surprising if there were enough remaining perceptual variance in ConceptNet after controlling for THINGS to produce such a large effect. This would be especially surprising given that ConceptNet was trained on text and correlates well with both explicit and implicit human semantic similarity judgments, whereas the THINGS dimensions were trained on data derived from visually presented objects. Moreover, perceptual similarity as assessed with the THINGS embedding space did predict performance quite well in the picture-cue condition, providing evidence that it is a reasonable proxy for the true perceptual similarity space.

It should be noted that the THINGS-based perceptual similarity between the target and distractor had little or no ability to predict performance for the first exposure in the category-cue condition. However, the lack of an effect of perceptual similarity in this case is not surprising given that our measure of perceptual similarity reflected the similarity between a specific target that participants had not yet seen and a specific distractor that they had not yet seen. That is, it seems unlikely that differences in features such as color and orientation between a particular target and a particular distractor could impact search performance when the participant had never before seen that target-distractor pair.

Participants presumably use their knowledge of the prototypical visual properties of the categories to guide search in tasks such as the present experiment. Unfortunately, we do not have a means of knowing the location of a general category (e.g., “grasshopper”) within the THINGS embedding space. This makes it impossible for us to test the hypothesis that participants used perceptual as well as conceptual features to guide attention on the first trial with a given category cue. Thus, although the present results provide strong evidence that participants did use conceptual information to guide attention in this case, we could not test whether they also used category-general perceptual information to guide attention (which seems likely).

## Experiment 2: The effect of perceptual and conceptual similarity on (non-target) dwell time

In Experiment 1, we investigated the ability of representational spaces with differing levels of conceptual and visual information to predict *attention-getting* behavior (i.e., the probability that gaze would shift to a given item). However, search also involves *attention-holding* mechanisms: once an item is fixated, the searcher must pause to decide whether the currently fixated object is indeed the target. In the literature on human development, there are well-established differences between these two processes. For example, Cohen (1972) found that attention-getting in infants was strongly determined by the physical salience of a stimulus whereas attention-holding depended on the complexity of a stimulus. In adults, recent work has demonstrated that the template for visual search contains coarser information for attention-getting behavior (or “search guidance”) compared to attention-holding behavior (or “target-match decisions”; Yu et al., 2022b).

Moreover, the search displays in Experiment 1 used only a set size of two, and the displays terminated after the first fixation, which is a very unusual type of visual search task. In Experiment 2, we increased the set size (to six) so that we could assess how different representational spaces predict search behavior when multiple items are searched on each trial.

**Fig. 4:**
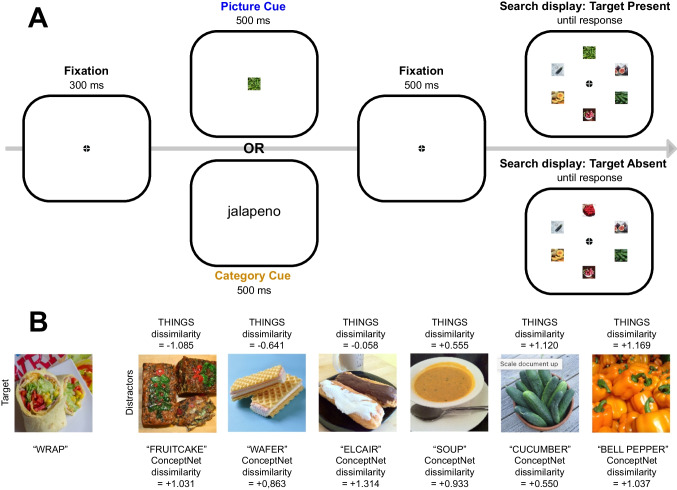
**A**) Sequence of events in Experiment 2. Stimuli are draw to scale. One group of participants saw picture cues and another group saw category cues. **B**) Example of target and distractor, including THINGS dissimilarity between the target image and each distractor image and ConceptNet dissimilarity between each target label and each distractor label

In Experiment 2, we used a more traditional present/absent search task with a set size of six items (Fig. 4A), and we focused on how the perceptual and conceptual properties of each item in the search display impacted attention-holding (the duration of fixation on that item). As in Experiment 1, each trial began with a picture cue in one group of participants or a category cue in another group of participants. Each search display contained six items distributed around a virtual circle. The array contained a target on 50% of trials, and participants were instructed to report target presence or absence by means of a speeded button press. There were 24 target objects, each associated with six distractors that varied in both their conceptual and their perceptual similarity to that specific target (Fig. 4B). Participants searched for each target 24 times.

Our primary analysis concerned the dwell time of gaze on distractor items. We focused on target-absent trials, because the dwell times on those trials could not be impacted by the simultaneous presence of the target and because many more distractors were fixated on target-absent trials than on target-present trials. We used an LME analysis that allowed us to determine the unique ability of each similarity space to predict distractor dwell time, controlling for the other space. We expected that if conceptual information can influence the target-decision stage of search, its effect would be most pronounced when searchers had minimal information about the specific visual properties of the target (i.e., on the first search for a given target when cued with a category label).

## Materials and methods

### Participants

We collected data from 48 new participants in Experiment 2 (picture-cue condition: 20 women, four men; category-cue condition: 18 women, six men). No participants were replaced for performance.

### Stimuli

In Experiment 2, our goal in creating the stimulus set was to find 24 targets from the THINGS database, with each target grouped with a set of six distractors that varied systematically in their similarity to the corresponding target. We needed six distractors for each target because the set size was six and there were target-absent trials that contained six items. To create such a stimulus set, we first selected the 40 most similar target-distractor pairs from Experiment 1, sorting them from most to least similar. We then selected five additional distractors to go along with each of the 40 targets. For a given target, we computed the Euclidean distance between the target and all possible images in the original set of 1,854 objects, and we created a temporary set of all images that had a similarity score (to the current target) less than 1.6. We then randomly chose five images from this temporary set to serve, along with the original distractor image from Experiment 1, as the distractors for this particular target image. We then repeated this process for each of the 40 target-distractor pairs, ensuring that the distractors selected for each target were different from all other target and distractor images. For some targets, not enough distractors were found that met these criteria, and these targets were dropped from the list of stimuli. This process concluded with 24 stimulus groups, consisting of a target with six unique distractors. At least one of the distractors was highly similar to the target (i.e., the distractor for the initial target-distractor pair), and the other distractors varied randomly in their similarity to the target. Figure 4B shows an example of one target and the associated distractors, along with the THINGS dissimilarity and ConceptNet dissimilarity between the target and each of the six distractors. The full list of target and distractor labels is provided in OSM Table S6 (sorted by THINGS dissimilarity) and Table S7 (sorted by ConceptNet dissimilarity).

As illustrated in Fig. 4A, the search display consisted of the central fixation dot along with either the target and five distractors (on target present trials) or six distractors (on target absent trials). The target and distractors were presented equidistant from each other on the same size virtual circle as in Experiment 1 (radius = 3.875°), with a random radial offset on each trial. The target and the distractor(s) each subtended 3.1° × 3.1°, and the center-to-center distance between one item and the adjacent items subtended 3.875°.

### Procedure

The sequence of events on a single trial is illustrated in Fig. 4 On each trial, the central fixation dot was presented for 300 ms, followed by a 500-ms cue, then a 500-ms central fixation dot, and then the search display along with the fixation dot. Participants were instructed to make a speeded button press response using one of two buttons on a gamepad for each search display to indicate whether the target for that trial was present or absent (left index finger for target-present, right index finger for target-absent). The search display and fixation dot terminated when the button was pressed. Incorrect responses triggered an “Incorrect!” message for 5 s in a red font, while correct responses ended the trial. The next trial began after a 400-ms inter-trial interval.

On target-absent trials, all six distractors associated with the cued target were presented in the search display. On target-present trials, a randomly selected five of the six distractors associated with the cued target were presented in the search display. Across the 12 target-absent searches for each target, each of the six distractors was left out from the display twice to equate experience with all images. Each target was cued 24 times (12 on target-present trials, 12 on target-absent trials) for 576 total trials, split into six blocks of 96 trials. The order of the 576 trials was randomized for each participant.

### Data analysis in Experiment 2

Our main analyses focused on the amount of time gaze dwelled on distractor items. Dwell time was calculated as the number of eye position samples (multiplied by 2, as the tracker sampled as 500 Hz) that occurred inside a circular area of interest (of radius 3.6°) around each picture in the search display. We included only dwell times longer than 75 ms so that eye-position samples from eye movements that traversed an object during the trajectory of an eye movement to a different object were not included in the analysis. In addition, we included data only from target-absent trials, which included many more distractor fixations. That is, because the target was found relatively quickly (mean response time (RT) = 1,214 ms, compared to 1,613 ms on target-absent trials), relatively few distractors were fixated on target-present trials (mean = 2.0, compared with a mean of 4.7 on target-absent trials). In addition, target-absent trials provide a purer measure of distractor processing because the presence of a target could influence the dwell time on a given distractor. For example, imagine a searcher is fixating a non-target item directly adjacent to the target. While fixating this item, the target could be identified in the periphery, leading to a relatively fast disengagement from the non-target. This process can never occur on target-absent trials, making target-absent trials a purer means of assessing attention-holding by distractors. Finally, the ability to predict target-absent performance is a major benefit of the present approach, as most computational models predict performance only for target-present trials (see, e.g., Adeli et al., 2017; however, cf. Mondal et al., 2023). The data from the target-present trials are available at https://osf.io/su9nf/ for anyone who wishes to examine eye-movement patterns on these trials.

The fixed- and random-effects structure of the models for Experiment 2 were identical to those of Experiment 1, except that the outcome variable was distractor dwell time, so a normal linear mixed effects model was used instead of a logistic model. Marginal effects were tested with Wald *t*-tests using degrees of freedom computed using Satterthwaite’s method (Kuznetsova et al., 2017).

In Experiment 1, our primary investigation was on saccade choice accuracy, with a supplementary analysis on the latency of the saccade toward the target. In Experiment 2, however, only the dwell time of each fixation could be validly analyzed, so we did not perform a corresponding analysis of the probability of fixating an item. One major problem with trying to analyze the probability of fixating an item is that the distance between items was no longer controlled after the first fixation, introducing substantial variation in the eccentricity of the items relative to the current point of fixation. This does not apply to the first saccade on each trial, but an analysis of the first saccade has a different problem: There was no specific incentive for making an accurate first eye movement. In fact, we found that most participants (46 out of 48) tended to make their initial saccade to a particular spatial region (e.g., the item that was closest to directly left of central fixation) on the vast majority of trials rather than being guided by the properties of the target and distractors.

## Results and discussion

### Overall performance across the 12 exposures

**Fig. 5:**
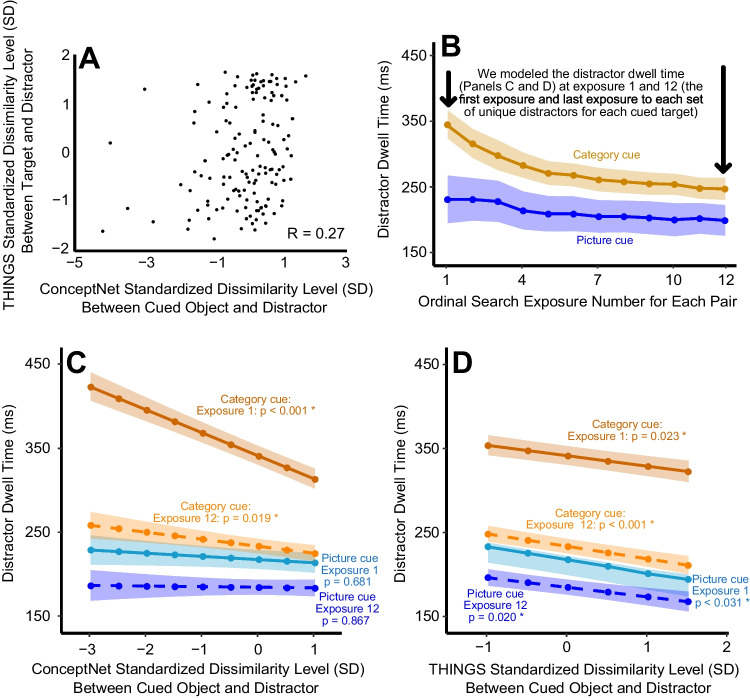
**A**) Scatterplot of the relationship between THINGS and ConceptNet standardized dissimilarity scores for the targets and associated distractor in the Experiment 2. **B**) Observed mean distractor dwell times across non-target exposures to each cue, separately for the category-label-cue and the picture-cue. **C** and **D**) Estimated distractor dwell times from the statistical model, showing for the effect of ConceptNet standardized dissimilarity (**C**) level or THINGS similarity level (**D**) between the cued target object and a given distractors (on target-absent trials). Note that both predictors were fit in the same model. Models fits are plotted separately for the category-label-cue and the pictured-cue conditions at Exposure 1 (the first non-target search for each pair) and Exposure 12 (the last non-target search for each pair). Shaded regions show ±1 standard error

Figure 5B depicts the observed results for the relationship between Exposure Number and distractor dwell time in Experiment 2, separately for the category-cue and the picture-cue conditions. At all Exposure Numbers, dwell times were longer for the category-cue than for the picture-cue. However, dwell times decreased more rapidly across exposures for the category-cue condition than for the picture-cue condition, and the difference between conditions became progressively smaller over exposures. As in Experiment 1, we included only the first and 12th target-absent exposures in the statistical analysis to avoid assuming a specific learning curve and to focus on comparing performance prior to any exposure to the specific targets with performance after substantial exposure. The data from all exposures are available at https://osf.io/su9nf/ for anyone who wants to model the learning process. The results for each individual exposure are shown in OSM Fig. S3.

### Effects of conceptual and perceptual similarity

The full statistical model output is provided in OSM Table S3. Figure 5C and D illustrate the model results for ConceptNet dissimilarity and THINGS dissimilarity, respectively (for a single model containing both variables).

#### Category-cue condition

For the first target-absent exposure of a category cue, distractor dwell time was strongly predicted by the conceptual dissimilarity between the cued target and the distractor. Specifically, a 1-SD increase in ConceptNet dissimilarity predicted a 27-ms decrease in mean distractor dwell time (*t*(177.3) = −5.72, *p* <.001). Perceptual dissimilarity between the cued target and distractor also predicted distractor dwell times, though the effect was relatively weak: a 1-SD increase in THINGS dissimilarity predicted only a 12-ms decrease in mean distractor dwell time (*t*(171.8) = −2.66, *p* =.009).

By the 12th target-absent exposure of a category cue, the relative strengths of the conceptual and perceptual effects reversed: A 1-SD increase in ConceptNet dissimilarity predicted only a 9-ms decrease in mean distractor dwell time (*t*(254.5) = −2.35, *p* =.020), whereas a 1-SD increase in THINGS dissimilarity predicted (similar to Exposure 1) a 15-ms decrease in mean distractor dwell times (*t*(225.8) = −4.38, *p* <.001). Note, however, that this difference between the relative strengths of the conceptual and perceptual effects is a qualitative description, and there is no perfect way to statistically compare them because we cannot assume that the range of dissimilarities was the comparable for the conceptual and perceptual spaces.

#### Picture-cue condition

The results for both the first and 12th exposures in the picture-cue condition were somewhat similar to the results from the 12th exposure in the category-cue condition. Specifically, THINGS dissimilarity significantly predicted distractor dwell times in the picture-cue condition (first exposure: *t*(205.9) = −3.05, *p* =.003, 1-SD increase in THINGS dissimilarity predicted a 15-ms decrease in mean distractor dwell times; 12th exposure: *t*(428.3) = −2.84, *p* =.005, 1-SD increase in THINGS dissimilarity predicted a 12-ms decrease in mean distractor dwell times), but ConceptNet dissimilarity did not (first exposure: *t*(224.7) = −0.67, *p* =.504, 1-SD increase in ConceptNet dissimilarity predicted a 3-ms decrease in mean distractor dwell times; 12th exposure: *t*(567.2) = −0.22, *p* =.826, 1-SD increase in ConceptNet dissimilarity predicted a 1-ms decrease in mean distractor dwell times).

#### Interactions:

The general finding that conceptual similarity mainly had an impact when the perceptual features of the target were not known (i.e., the first exposure in the category-cue condition) was supported by a significant three-way interaction between ConceptNet dissimilarity, Cuing Condition, and Exposure Number (*t*(8493) = −2.72*, p* =.006). Note that this pattern developed gradually across exposures (see OSM Fig. S3). By contrast, the effects of perceptual similarity remained relatively constant across conditions, with only a significant main effect of THINGS dissimilarity but no significant three-way interaction of THINGS dissimilarity, Cuing Condition, and Exposure Number. Again, this is a qualitative difference between the effects of ConceptNet dissimilarity and THINGS dissimilarity.

#### Summary

When searchers knew only the category of the cued target and not its specific visual features, the amount of time the eyes remained fixed on a given distractor object was strongly predicted by the conceptual relatedness between the cued target and the distractor (ConceptNet), whereas the perceptual relatedness between the cued target and the distractor had relatively little effect (THINGS). However, the role of conceptual similarity became much weaker when the searchers had detailed visual information about the target (either by being provided with an exact picture of the target or by having many exposures to a particular exemplar for each unique category). Thus, like the probability of fixation in Experiment 1, the dwell time of attention on a given item is predicted by conceptual information when the perceptual details of the target are not known, but perceptual information is the main driver of dwell times when the searcher knows exactly what the target looks like.

## General discussion

In the present experiments, we sought to assess the extent to which people use conceptual and perceptual information when they search for real-world objects. To that end, we simultaneously quantified the conceptual and perceptual dissimilarity between the search target and the distractor(s) in our search tasks. The key results came from the first exposure in the category-cue condition, when the searchers had never seen the specific target and distractor(s). Under these conditions, which resemble many real-world searches, searchers would be maximally motivated to use conceptual information to guide search.

### Key findings

On the first exposure in the category-cue condition of Experiment 1, we found that the guidance operation of search was strongly predicted by the conceptual relatedness between the target and distractor. In particular, saccades were more often directed toward the distractor instead of the target as the distractor became more and more conceptually similar to the target. The statistical analysis controlled for the perceptual similarity between the target and the distractor, so this result cannot be easily explained by correlations between conceptual and perceptual similarity. On the first exposure in the category-cue condition of Experiment 2, we found that the duration of the target decision operation that occurs after an object is fixated was also predicted by the conceptual relatedness of the target and distractor. In particular, distractors tended to be fixated longer the more conceptually similar they were to the target on the first exposure in the category-cue condition. Again, this effect was observed after statistically controlling for perceptual similarity. Together, these results provide new evidence that conceptual information can influence visual search.

After the first exposure to the specific target and distractor associated with a given category label, search performance became increasingly predicted by the perceptual similarity between the target and the distractor and less predicted by their conceptual similarity. Thus, after only a few trials of search repetition, searchers can learn the specific visual properties of a category exemplar and use those properties to guide attention. This is especially impressive given that searchers were learning 80 different cue-target pairs in Experiment 1 and 24 different cue-target pairs in Experiment 2. In other words, searchers can learn the specific features associated with many different labels, and this learning is quite rapid.

### Mechanisms of conceptual guidance

How might conceptual information influence the search process? The explanation seems straightforward for the target decision operation that occurs once an object is fixated. In this case, fixating a particular object allows searchers to form a coherent, meaningful object representation that presumably makes contact with high-level semantic information (as in many models of attention, from Feature Integration Theory – Treisman & Gelade, 1980 — to the most recent instantiations of Guided Search – Wolfe, 2021). Thus, conceptual information is readily available to impact the decision about whether the fixated object matches the category label given at the beginning of the trial. However, it was not a foregone conclusion that this information would actually impact the distractor dwell times. For example, in a search for a jalapeno, an eggplant distractor had above-average conceptual similarity but below-average perceptual similarity. However, an eggplant is clearly not a jalapeno, so during a search for a jalapeno, it is not inevitable that attention would dwell longer on an eggplant than on a more conceptually distant object such as a cigar.

**Fig. 6:**
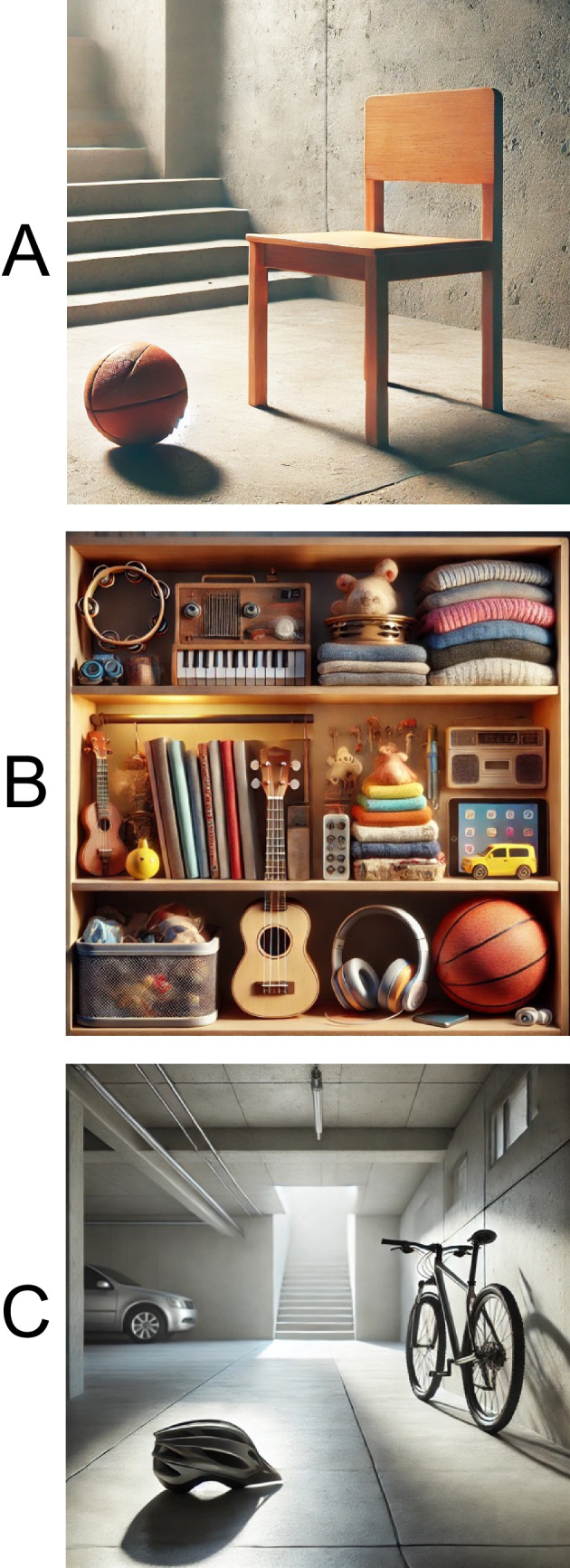
Example of real-worl visual search situations. **A**) Someone is searching for a pair of athletic shoes, and the conceptually related basketball could easily be perceived without a shift of attention given the lack of clutter in the scene. **B**) Someone is searching for a pair of athletic shoes, and the conceptually related basketball is unlikely to be perceived without a shift of attention given the clutter in the scene. **C**) Someone is searching for a bike helmet in a garage, and both the conceptually related bicycle and the conceptually unrelated car could easily be perceived without a shift of attention given the lack of clutter in the scene

Whereas the role of conceptual information in the target decision operation is compatible with most models of visual search (because the target is already attended at this point), it is less obvious how conceptual information could influence the search guidance operation that occurs prior to shifts of attention. We propose a mechanism inspired by Treisman’s classic attenuator model of attention (Treisman, 1960). Consistent with the evidence described in the *Introduction*, we propose that information about object identity (including contact with semantic information) occurs preattentively across all objects in a degraded (attenuated) but above-chance manner (as opposed to occurring only inside the focus of attention). This coarse information can then be used to guide attention toward objects that match an abstract conceptual target category. Depending on factors such as the number of objects in the display, their eccentricity, and the degree of crowding, this guidance might be quite good, moderate, or quite poor. For example, in a relatively empty room containing a chair and a basketball, the basketball could easily activate a conceptual representation prior to being fixated, and it might attract attention if someone was searching for a pair of athletic shoes (see Fig. 6A). However, the same basketball would be unlikely to be identified and attract attention if it was on a crowded shelf with dozens of other objects (see Fig. 6B). Experiment 1 of the present study involved displays containing only two items, and although the items were relatively small, it should be unsurprising that the visual system could rapidly extract some identity information about both items and use this information to guide attention.

### Covert versus overt attention

The present study examined overt shifts of gaze and did not attempt to separately measure shifts of covert attention. It is therefore important to ask whether the observed effects of conceptual information on the guidance of overt attention in Experiment 1 might actually reflect covert attentional processes that preceded the shifts of gaze. In other words, it seems possible that only perceptual information can guide covert attention, and that the effects of conceptual information on overt attention arose from conceptual information that was obtained as a result of covert shifts of attention.

For example, imagine a trial in Experiment 1 on which preattentive perceptual information was insufficient to guide either covert or overt attention to the target. In such a case, covert attention might shift to one of the two items at random. This would allow that item to be identified, activating a conceptual representation. If the match between this conceptual representation and the category label was good, gaze could then be shifted to that item. If the match was poor, then gaze could be shifted to the other item. Consistent with this possibility, saccade latencies were quite long on Exposure 1 for the category-cue condition (see Fig. S1, OSM), providing time for one to two shifts of covert attention prior to the eye movement.

Although we cannot conclusively rule out this possibility, it does not seem very plausible upon a more careful examination. For example, imagine that the participant is searching for the bike helmet in a garage that also contains a bicycle and a car (Fig. 6C). The bicycle is conceptually related to the bike helmet but visually quite different. If covert attention shifts to the bicycle, allowing enough information to be extracted to determine that it is conceptually related to the bike helmet, this would presumably be enough information to know that it is not a bike helmet. Why, then, would participants be more likely to shift gaze to the bicycle in a search like this than to shift gaze to the conceptually unrelated car? This seems implausible, but we cannot conclusively rule it out on the basis of the current data, as we have no direct measure of covert attention. Future studies could assess the role of covert attention in a design like the present experiment by measuring, for example, the N2pc component (Luck, 2012).

### Consistent target-distractor pairs/sets and their influence on search behavior

In Experiment 1, a given target object was always presented with the same distractor object. Similarly, in Experiment 2, each search for a given target was always presented with five distractors from the same set of six possible distractor objects. Such consistency between the target and distractor(s) is known to influence search. Specifically, searchers learn which features maximally distinguish the target from its associated distractor(s) and use this information to modify the target template (Navalpakkam & Itti, 2007; Yu et al., 2022a). It is entirely possible that participants in the present study also learned to use information about the distractors associated with a given target to guide their search behavior. However, because we did not vary the target-distractor combinations, we have no way of determining whether this actually occurred. This would be an interesting topic for future work.

### Relationship to previous studies of conceptual guidance

Other researchers have also assessed the role of conceptual information in influencing search. For example, two studies (de Groot, et al., 2016; Nuthmann, et al., 2019) investigated the role of capture during visual search by distracting stimuli that *either* matched the target’s conceptual category but not it’s perceptual features (the “semantic competitor”) *or* matched the target’s perceptual features but not it’s conceptual category (the “visual competitor”). These studies consistently found that both the semantic competitor and the visual competitor captured attention more strongly than a distractor that was neither conceptually nor visually related to the target (the “unrelated competitor”).

Although the studies did an admirable job of controlling their stimuli, full control may have been impossible given the design of the studies. As previously discussed, stimuli that belong to the same/similar conceptual category will be more perceptually similar to one another (on average) than to stimuli belonging to different/dissimilar categories. Indeed, the “visual competitors” in these studies were (on average) more closely related conceptually to the target than the “unrelated competitors.” Likewise, the “semantic competitors” were (on average) more closely related visually to the target than were the unrelated competitors. Thus, the greater capture of attention by semantic competitors than unrelated competitors may have been due to the greater perceptual similarity of the semantic competitors to the target. This highlights the usefulness of the methodological and analytic approach introduced in the present study: by simultaneously considering the perceptual and conceptual relatedness of the distractor(s) to the target, we were able to statistically control for the other factor, ruling out that our effects of conceptual relatedness were actually due to perceptual relatedness.

Two other studies (Malcolm et al., 2016; Nah et al., 2021) have convincingly demonstrated that conceptual information can influence attention. In general, these experiments found that attention spread toward peripheral stimuli more for peripheral stimuli that matched the category of the currently attended item than for peripheral stimuli that did not match that category. However, these studies were not search tasks and were not directly interested in either the goal-directed guidance of attention or post-target decision-making in search. Instead, they probed the relatively automatic spread of spatial attention toward locations/objects that match currently attended locations/objects. Nonetheless, the results of these studies were broadly convergent with the results of the present study.

### Summary and future directions

The present study provided strong evidence that overt attention can be guided by conceptual information when participants are cued to the category of a visual search target and have never seen the actual target before. When they gain experience with the specific instance of that category (either by searching for the same instance multiple times or being shown a picture as a cue), participants become increasingly reliant on perceptual information and less reliant on conceptual information. This matches the ecological reality of how humans search the environment in the natural world: they sometimes know only the category of the search target and sometimes have experience with the specific visual features of the target.

However, several open questions remain. First, can covert attention also be controlled by conceptual information, or is covert attention limited to guidance from visual information, as proposed by the version 6 of the guided search theory (Wolfe, 2021)? Second, are there perceptual features that are missing from the THINGS space or conceptual features that are missing from the ConceptNet space that are used in guiding attention? Third, as people gain experience with a given search task, how is search behavior impacted by knowledge of the target versus knowledge of the distractor? Finally, if one distractor in a search array is conceptually distinct from all the others, can this “conceptual singleton” capture attention or be suppressed in a manner similar to singletons defined by low-level features (Gaspelin & Luck, 2018; Pashler, 1988; Sawaki & Luck, 2010; Theeuwes, 1993; Yantis & Jonides, 1984)? By quantifying both perceptual and conceptual similarity and modeling both simultaneously, as we have done in the present study, future research should be able to answer these questions.

## Supplementary Information

Below is the link to the electronic supplementary material.

Supplementary file1 (PDF 54.6 KB)

Supplementary file2 (PDF 65.4 KB)

Supplementary file3 (PDF 65.4 KB)

Supplementary file4 (PDF 55.6 KB)

Supplementary file5 (PDF 56.5 KB)

Supplementary file6 (PDF 83.8 KB)

Supplementary file7 (PDF 84.1 KB)

Supplementary file8 (PDF 63.1 KB)

Supplementary file9 (PDF 58.3 KB)

Supplementary file10 (PDF 57.5 KB)

Supplementary file11 (PDF 5.20 MB)

Supplementary file12 (PDF 5.20 MB)

## Data Availability

All data and materials for all experiments are available via the Open Science Framework at https://osf.io/su9nf/

## Footnotes

1 It is well established that search can be guided by *scene-level* semantics. For example, search for a coffee mug in a kitchen is strongly restricted to those regions of the scene likely to contain coffee mugs (mid-level flat surfaces). Here, we are interested in whether the semantics of *objects* can also guide attention to those objects.

2 The present study focused on overt attention rather than covert attention because it is much easier to directly assess where overt attention is focused. The *General discussion* considers whether our results would also apply to covert attention.

## Code availability

All analysis and experimental control code is available via the Open Science Framework at https://osf.io/su9nf/
