# Supplementary material for: Combined conceptual and perceptual control of visual attention in search for real-world objects
Source: Atten Percept Psychophys. 2025 Sep 25;88(2):59. doi: 10.3758/s13414-025-03116-4 (PMC12864220; doi:10.3758/s13414-025-03116-4)
Supplement: Supplementary file 1 — Supplementary file1 (PDF 54.6 KB) [file 13414_2025_3116_MOESM1_ESM.pdf]

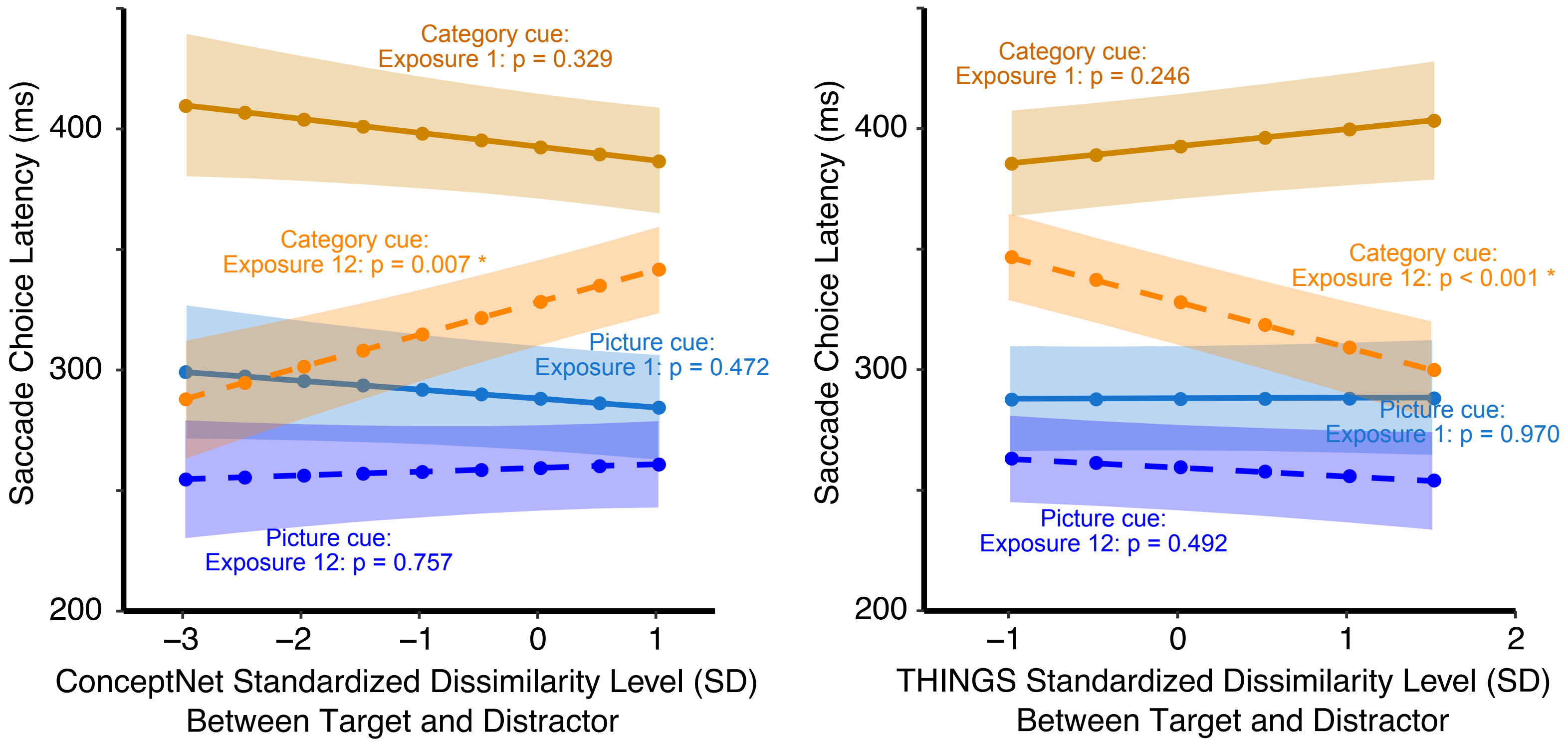

Supplementary Figure S1. A and **B)** Linear mixed effect model fits for the effect of standardized (z-scored) THINGS dissimilarity level **(A)** or ConceptNet dissimilarity level **(B)** between target-distractor pairs on saccade choice latency. Model fits are plotted separately for the category-label-cue and the picture-cue conditions at Exposure 1 (the first search for a given pair) and Exposure 12 (the last search for a given pair).
