# Supplementary material for: Combined conceptual and perceptual control of visual attention in search for real-world objects
Source: Atten Percept Psychophys. 2025 Sep 25;88(2):59. doi: 10.3758/s13414-025-03116-4 (PMC12864220; doi:10.3758/s13414-025-03116-4)
Supplement: Supplementary file 2 — Supplementary file2 (PDF 65.4 KB) [file 13414_2025_3116_MOESM2_ESM.pdf]

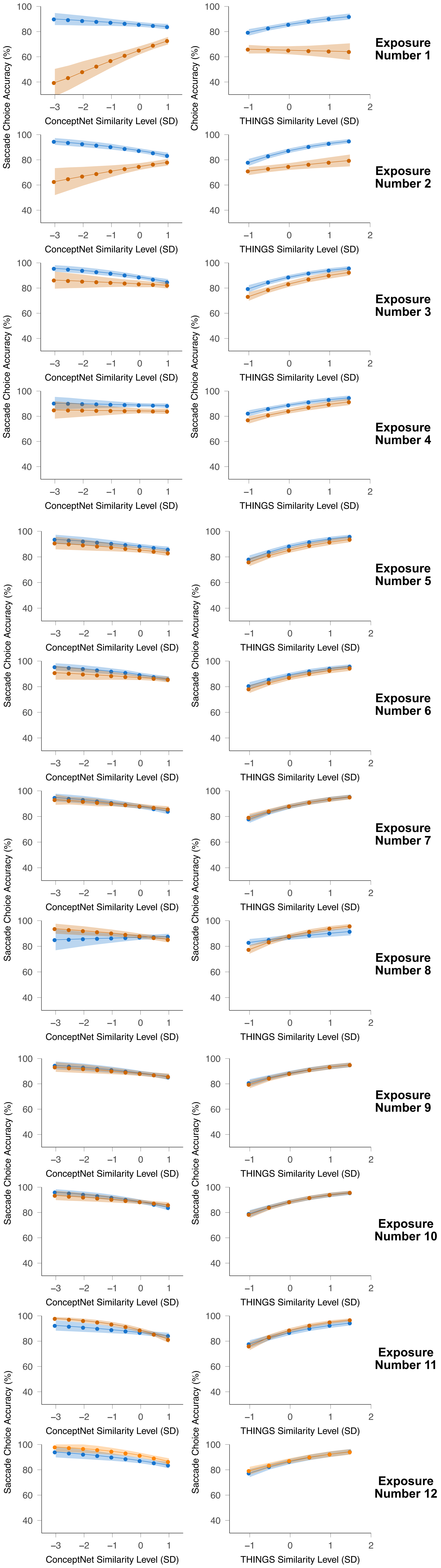

Supplementary Figure S2. Generalized linear model fits for the effect of ConceptNet standardized dissimilarity level (left) or THINGS dissimilarity level (right) between target and distractor pairs on saccade choice accuracy. Model fits are plotted separately for category cues (orange) and picture cues (blue) at each each ordinal Exposure Number (1 through 12). For the category cues, the effect of ConceptNet dissimilarity on saccade choice accuracy was stronger than the effect of THINGS dissimilarity at the early exposures.
