# Supplementary material for: Combined conceptual and perceptual control of visual attention in search for real-world objects
Source: Atten Percept Psychophys. 2025 Sep 25;88(2):59. doi: 10.3758/s13414-025-03116-4 (PMC12864220; doi:10.3758/s13414-025-03116-4)
Supplement: Supplementary file 3 — Supplementary file3 (PDF 65.4 KB) [file 13414_2025_3116_MOESM3_ESM.pdf]

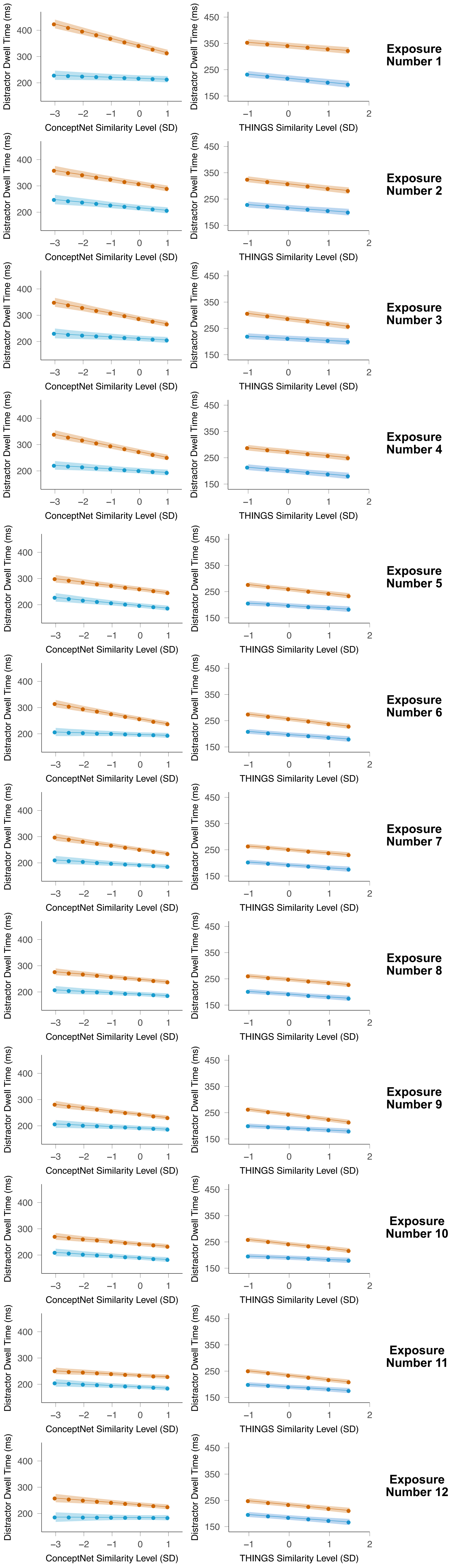

Supplementary Figure S3. Generalized linear model fits for the effect of ConceptNet standardized dissimilarity level (left) or THINGS dissimilarity level (right) between the cued target object and distractor on distractor dwell times. Model fits are plotted separately for the category-label-cue (orange) and the picture-cue condition (blue) at each each ordinal Exposure Number (1 through 12).
