# Supplementary material for: Combined conceptual and perceptual control of visual attention in search for real-world objects
Source: Atten Percept Psychophys. 2025 Sep 25;88(2):59. doi: 10.3758/s13414-025-03116-4 (PMC12864220; doi:10.3758/s13414-025-03116-4)
Supplement: Supplementary file 5 — Supplementary file5 (PDF 56.5 KB) [file 13414_2025_3116_MOESM5_ESM.pdf]

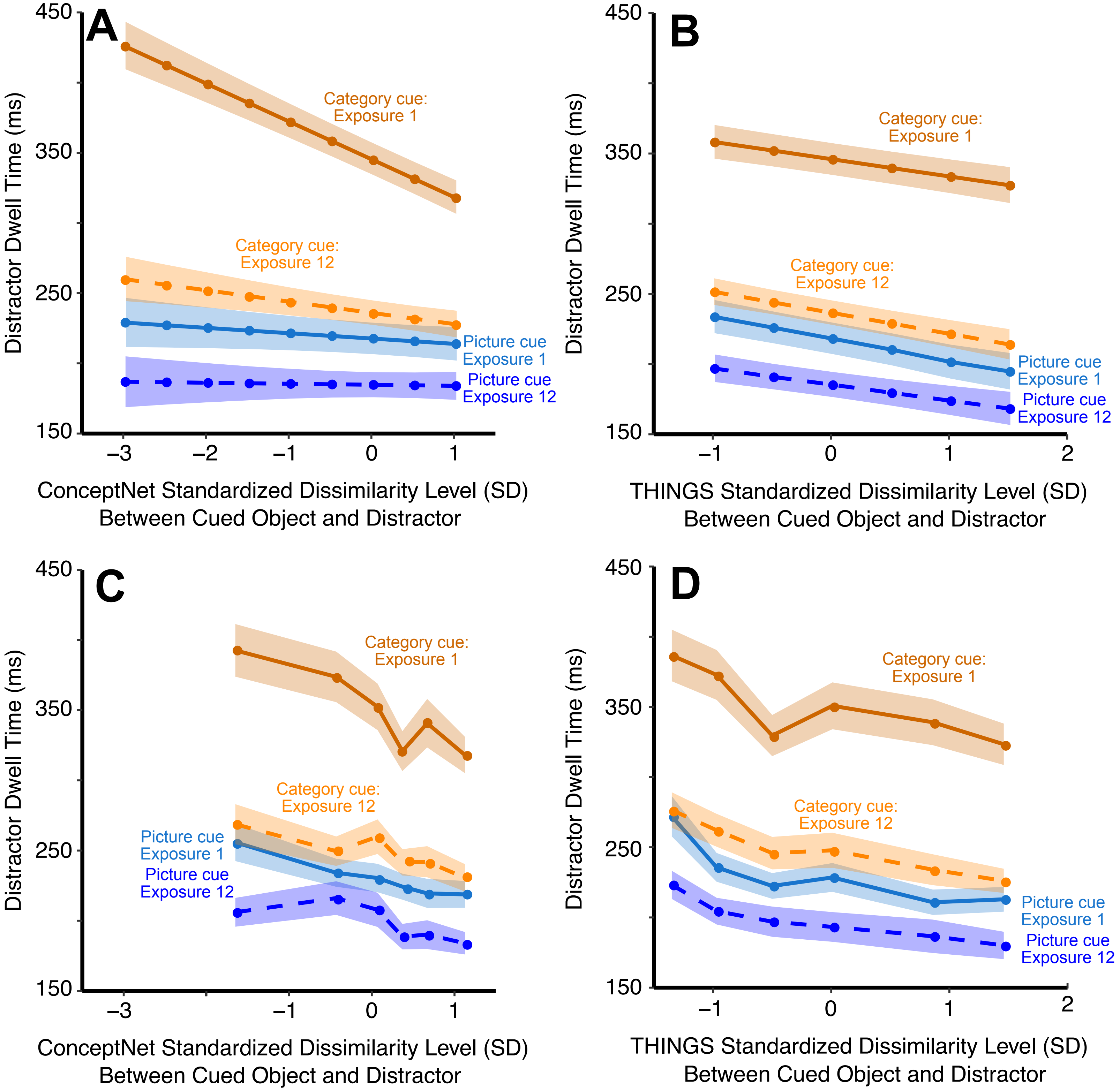


**Supplementary Figure S5. A and B)** Linear model fits for the effect of ConceptNet standardized dissimilarity **(A)** level or THINGS dissimilarity level **(B)** between the cued target object and distractor on distractor dwell times (on Target Absent trials). Model fits are plotted separately for the category-label-cue and the picture-cue conditions at Ordinal Search Exposure 1 (the first search for each pair) or Ordinal Search Exposure 12 (the last search for each pair). **C and D)** Mean observed data for the effect of ConceptNet standardized dissimilarity **(C)** level or THINGS dissimilarity level **(D)** between the cued target object and distractor on distractor dwell times (on Target Absent trials). Model fits are plotted separately for the category-label-cue and the picture-cue conditions at Ordinal Search Exposure 1 (the first search for each pair) or Ordinal Search Exposure 12 (the last search for each pair).
