# Supplementary material for: Combined conceptual and perceptual control of visual attention in search for real-world objects
Source: Atten Percept Psychophys. 2025 Sep 25;88(2):59. doi: 10.3758/s13414-025-03116-4 (PMC12864220; doi:10.3758/s13414-025-03116-4)
Supplement: Supplementary file 6 — Supplementary file6 (PDF 83.8 KB) [file 13414_2025_3116_MOESM6_ESM.pdf]

Fixed Effects Structure for:

Accuracy ~ THINGS_Sim * ConceptNet_Sim * Position_0 * Condition + (1 + Position_0 | SubNum) + (1 + Position_0 | ItemNum)

Predictor(s)	Estimate	Std. Error	*z*	*p*
Intercept	0.63	0.14	4.52	< 0.001
THINGS Similarity	-0.03	0.14	-0.26	0.798
ConceptNet Similarity	0.35	0.13	2.72	0.007
Exposure Number	1.29	0.17	7.47	< 0.001
Cuing Cuing Condition	1.16	0.18	6.59	< 0.001
THINGS_Similarity:ConceptNet_Similarity	0.26	0.13	2.09	0.037
THINGS_Similarity:Exposure Number	0.61	0.17	3.58	< 0.001
ConceptNet_Similarity:Exposure Number	-0.63	0.16	-3.91	< 0.001
THINGS_Similarity:Cuing Condition	0.47	0.15	3.11	0.002
ConceptNet_Similarity:Cuing Condition	-0.49	0.14	-3.37	< 0.001
Exposure Number:Cuing Condition	-1.21	0.24	-5.06	< 0.001
THINGS_Similarity:ConceptNet_Similarity:Exposure Number	-0.34	0.16	-2.11	0.035
THINGS_Similarity:ConceptNet_Similarity:Cuing Condition	-0.41	0.14	-2.91	0.004
THINGS_Similarity:Exposure Number:Cuing Condition	-0.41	0.24	-1.74	0.081
ConceptNet_Similarity:Exposure Number:Cuing Condition	0.55	0.22	2.45	0.014
THINGS_Similarity:ConceptNet_Similarity:Exposure Number:Cuing Condition	0.30	0.22	1.39	0.165

Supplementary Table 1. Overall statistical model for Saccade Choice Accuracy in Experiment 1. Shaded rows correspond to statistically significant results. Note the model (as fit in R; see main text) is listed above the table.
