# Supplementary material for: Combined conceptual and perceptual control of visual attention in search for real-world objects
Source: Atten Percept Psychophys. 2025 Sep 25;88(2):59. doi: 10.3758/s13414-025-03116-4 (PMC12864220; doi:10.3758/s13414-025-03116-4)
Supplement: Supplementary file 7 — Supplementary file7 (PDF 84.1 KB) [file 13414_2025_3116_MOESM7_ESM.pdf]

Fixed Effects Structure for:

SaccadeLatency ~ THINGS_Sim * ConceptNet_Sim * Position_0 * Condition + (1 + Position_0 | SubNum) + (1 | ItemNum)

Predictor(s)	Estimate	Std. Error	df	*t*	*p*
Intercept	392.70	21.72	48.5	18.08	< .001
THINGS Similarity	7.14	6.15	671.0	1.16	0.246
ConceptNet Similarity	-5.75	5.89	687.3	-0.98	0.329
Exposure Number	-104.53	30.52	47.2	-3.43	< .001
Cuing Condition	-5.87	2.33	48.3	-2.52	0.015
THINGS_Similarity:ConceptNet_Similarity	-7.25	5.53	645.8	-1.31	0.191
THINGS_Similarity:Exposure Number	-6.93	7.45	5633.5	-0.93	0.352
ConceptNet_Similarity:Exposure Number	2.06	7.10	5632.9	0.29	0.772
THINGS_Similarity:Cuing Condition	-2.35	0.67	5629.1	-3.52	< .001
ConceptNet_Similarity:Cuing Condition	1.75	0.64	5632.6	2.74	0.006
Exposure Number:Cuing Condition	3.26	3.29	47.9	0.99	0.327
THINGS_Similarity:ConceptNet_Similarity:Exposure Number	7.30	6.71	5626.4	1.09	0.276
THINGS_Similarity:ConceptNet_Similarity:Cuing Condition	1.79	0.60	5629.0	2.98	0.003
THINGS_Similarity:Exposure Number:Cuing Condition	2.00	0.91	5609.9	2.20	0.028
ConceptNet_Similarity:Exposure Number:Cuing Condition	-1.27	0.86	5611.5	-1.47	0.141
THINGS_Similarity:ConceptNet_Similarity:Exposure Number:Cuing Condition	-1.48	0.82	5609.3	-1.81	0.071

Supplementary Table 2. Overall statistical model for Saccade Choice Latency in Experiment 1. Shaded rows correspond to statistically significant results. Note the model (as fit in R; see main text) is listed above the table.
