# Supplementary material for: Combined conceptual and perceptual control of visual attention in search for real-world objects
Source: Atten Percept Psychophys. 2025 Sep 25;88(2):59. doi: 10.3758/s13414-025-03116-4 (PMC12864220; doi:10.3758/s13414-025-03116-4)
Supplement: Supplementary file 8 — Supplementary file8 (PDF 63.1 KB) [file 13414_2025_3116_MOESM8_ESM.pdf]

Fixed Effects Structure for:

DwellTime ~ THINGS_Sim * ConceptNet_Sim * Position_0 * Condition + (1 + Position_0 | SubNum) + (1 | ItemNum)

Predictor(s)	Estimate	Std. Error	df	*t*	*p*
Intercept	341.39	11.57	58.6	29.51	< .001
THINGS_Sim	-12.25	4.61	171.8	-2.66	0.009
ConceptNet_Sim	-27.36	4.78	177.3	-5.72	< .001
Position_011	-106.39	8.93	49.9	-11.91	< .001
Condition	-124.52	15.42	46.7	-8.08	< .001
THINGS_Sim:ConceptNet_Sim	-3.44	4.38	179.1	-0.78	0.434
THINGS_Sim:Position_011	-2.58	3.93	251.9	-0.66	0.512
ConceptNet_Sim:Position_011	18.84	4.20	283.0	4.49	< .001
THINGS_Sim:Condition	-2.50	3.51	8462.8	-0.71	0.477
ConceptNet_Sim:Condition	23.95	3.80	8449.9	6.30	< .001
Position_011:Condition	76.70	12.63	49.9	6.07	< .001
THINGS_Sim:ConceptNet_Sim:Position_011	4.76	3.86	287.2	1.23	0.219
THINGS_Sim:ConceptNet_Sim:Condition	12.22	3.56	8477.6	3.43	< .001
THINGS_Sim:Position_011:Condition	5.70	5.43	8468.8	1.05	0.294
ConceptNet_Sim:Position_011:Condition	-16.44	6.04	8493.0	-2.72	< .001
THINGS_Sim:ConceptNet_Sim:Position_011:Condition	-12.30	5.67	8198.2	-2.17	< .001
