# Supplementary material for: Combined conceptual and perceptual control of visual attention in search for real-world objects
Source: Atten Percept Psychophys. 2025 Sep 25;88(2):59. doi: 10.3758/s13414-025-03116-4 (PMC12864220; doi:10.3758/s13414-025-03116-4)
Supplement: Supplementary file 9 — Supplementary file9 (PDF 58.3 KB) [file 13414_2025_3116_MOESM9_ESM.pdf]

Supplementary Table S4. Stimulus pairs (and associated dissimilarity scores for each) sorted by increasing THINGS dissimilarity

Label for Target	Label for Distractor	THINGS Dissimilarity	ConceptNet Dissimilarity
leopard	panther	-1.119	-3.295
casserole	egg roll	-1.104	-1.389
gazelle	rabbit	-1.063	0.057
jalapeno	zucchini	-1.038	-1.361
dog	tiger	-1.036	0.142
orangutan	sloth	-1.011	0.004
grasshopper	praying mantis	-0.987	-1.351
jeans	pants	-0.974	-3.637
fast food	stir fry	-0.973	-0.661
pony	lion	-0.970	0.194
calzone	croissant	-0.967	-1.168
scallop	corden bleu	-0.962	-0.745
quiche	lasagna	-0.954	-2.742
dip	scrambled egg	-0.952	0.245
llama	bison	-0.948	-0.096
cheetah	rat	-0.947	0.254
hamster	warthog	-0.940	0.358
baklava	ravioli	-0.938	-0.915
wrap	fruitcake	-0.937	0.710
gorilla	chinchilla	-0.936	0.348
guinea pig	boar	-0.892	-0.066
duckling	chipmunk	-0.843	0.095
grape	lime	-0.830	-0.037
breakfast	brownie	-0.823	-0.366
iguana	possum	-0.803	-0.569
turtle	anteater	-0.770	-0.159
tortilla	donut	-0.687	-0.648
cereal	enchilada	-0.672	-0.069
deer	mongoose	-0.672	0.457
lamp chop	chocolate	-0.652	-0.030
nacho	cornbread	-0.647	-0.451
ostrich	yak	-0.635	0.176
mole	zebra	-0.632	0.999
kitten	ram	-0.626	1.033
scone	cookie	-0.623	-1.860
pecan	grits	-0.612	-0.487
frog	bee	-0.590	-0.600
cougar	gopher	-0.560	-0.478
vegetable	squash	-0.547	-2.171
baby cow	turkey	-0.542	0.543
toast	vacuum	-0.073	0.319
ready meal	grapefruit	-0.057	0.793
ramp	turnstile	-0.048	-0.396
spaghetti	coleslaw	-0.042	-0.727
noodles	cashew	-0.025	-0.144
cardinal	hawk	-0.008	0.755
notepad	paper	0.003	-1.193
license plate	odometer	0.018	0.129
snowplow	trolley	0.032	-0.456
soy sauce	buffet	0.103	0.672
ship	fishing pole	0.118	0.633
pelican	bear	0.122	0.183
touchpad	springboard	0.146	0.313
pigeon	butterfly	0.157	-0.021
pantsuit	tag	0.168	0.990
shoe	bra	0.175	0.048
magnifier	webcam	0.197	-0.432
ginger	crème brulee	0.209	-0.303
necktie	neck	0.221	-0.740
shoulder	polo shirt	0.223	0.761
beard	inkwell	1.581	1.001
fountain pen	school bus	1.582	0.832
swimsuit	gear	1.583	0.300
honey	doorhandle	1.583	1.000
yoke	envelope	1.584	0.861
cheeseburger	train set	1.584	1.078
burrito	barbell	1.585	0.460
ice cream cone	wire cutters	1.585	0.608
curry	hoe	1.586	1.246
earplug	wand	1.587	0.764
crackers	spareribs	1.587	0.851
kite	compass	1.588	0.654
banana peel	volleyball	1.588	0.829
ear	waterwheel	1.589	1.122
iron	crepe	1.595	0.887
lizard	biscuit	1.595	1.098
siren	leggings	1.595	1.257
crystal	handbrake	1.596	1.562
coat	dragonfly	1.600	1.108
clam	tape	1.600	1.034
