# Supplementary material for: Combined conceptual and perceptual control of visual attention in search for real-world objects
Source: Atten Percept Psychophys. 2025 Sep 25;88(2):59. doi: 10.3758/s13414-025-03116-4 (PMC12864220; doi:10.3758/s13414-025-03116-4)
Supplement: Supplementary file 10 — Supplementary file10 (PDF 57.5 KB) [file 13414_2025_3116_MOESM10_ESM.pdf]

Supplementary Table S5. Stimulus pairs (and associated dissimilarity scores for each) sorted by increasing ConceptNet dissimilarity

Label for Target	Label for Distractor	THINGS Dissimilarity	ConceptNet Dissimilarity
jeans	pants	-0.974	-3.637
leopard	panther	-1.119	-3.295
quiche	lasagna	-0.954	-2.742
vegetable	squash	-0.547	-2.171
scone	cookie	-0.623	-1.860
casserole	egg roll	-1.104	-1.389
jalapeno	zucchini	-1.038	-1.361
grasshopper	praying mantis	-0.987	-1.351
notepad	paper	0.003	-1.193
calzone	croissant	-0.967	-1.168
baklava	ravioli	-0.938	-0.915
scallop	corden bleu	-0.962	-0.745
necktie	neck	0.221	-0.740
spaghetti	coleslaw	-0.042	-0.727
fast food	stir fry	-0.973	-0.661
tortilla	donut	-0.687	-0.648
frog	bee	-0.590	-0.600
iguana	possum	-0.803	-0.569
pecan	grits	-0.612	-0.487
cougar	gopher	-0.560	-0.478
snowplow	trolley	0.032	-0.456
nacho	cornbread	-0.647	-0.451
magnifier	webcam	0.197	-0.432
ramp	turnstile	-0.048	-0.396
breakfast	brownie	-0.823	-0.366
ginger	crème brulee	0.209	-0.303
turtle	anteater	-0.770	-0.159
noodles	cashew	-0.025	-0.144
llama	bison	-0.948	-0.096
cereal	enchilada	-0.672	-0.069
guinea pig	boar	-0.892	-0.066
grape	lime	-0.830	-0.037
lamp chop	chocolate	-0.652	-0.030
pigeon	butterfly	0.157	-0.021
orangutan	sloth	-1.011	0.004
shoe	bra	0.175	0.048
gazelle	rabbit	-1.063	0.057
duckling	chipmunk	-0.843	0.095
license plate	odometer	0.018	0.129
dog	tiger	-1.036	0.142
ostrich	yak	-0.635	0.176
pelican	bear	0.122	0.183
pony	lion	-0.970	0.194
dip	scrambled egg	-0.952	0.245
cheetah	rat	-0.947	0.254
swimsuit	gear	1.583	0.300
touchpad	springboard	0.146	0.313
toast	vacuum	-0.073	0.319
gorilla	chinchilla	-0.936	0.348
hamster	warthog	-0.940	0.358
deer	mongoose	-0.672	0.457
burrito	barbell	1.585	0.460
baby cow	turkey	-0.542	0.543
ice cream cone	wire cutters	1.585	0.608
ship	fishing pole	0.118	0.633
kite	compass	1.588	0.654
soy sauce	buffet	0.103	0.672
wrap	fruitcake	-0.937	0.710
cardinal	hawk	-0.008	0.755
shoulder	polo shirt	0.223	0.761
earplug	wand	1.587	0.764
ready meal	grapefruit	-0.057	0.793
banana peel	volleyball	1.588	0.829
fountain pen	school bus	1.582	0.832
crackers	spareribs	1.587	0.851
yoke	envelope	1.584	0.861
iron	crepe	1.595	0.887
pantsuit	tag	0.168	0.990
mole	zebra	-0.632	0.999
honey	doorhandle	1.583	1.000
beard	inkwell	1.581	1.001
kitten	ram	-0.626	1.033
clam	tape	1.600	1.034
cheeseburger	train set	1.584	1.078
lizard	biscuit	1.595	1.098
coat	dragonfly	1.600	1.108
ear	waterwheel	1.589	1.122
curry	hoe	1.586	1.246
siren	leggings	1.595	1.257
crystal	handbrake	1.596	1.562
