# Supplementary material for: Combined conceptual and perceptual control of visual attention in search for real-world objects
Source: Atten Percept Psychophys. 2025 Sep 25;88(2):59. doi: 10.3758/s13414-025-03116-4 (PMC12864220; doi:10.3758/s13414-025-03116-4)
Supplement: Supplementary file 12 — Supplementary file12 (PDF 5.20 MB) [file 13414_2025_3116_MOESM12_ESM.pdf]

Supplementary Table 7: Stimuli for Experiment 2. Item pairs are sorted by increasing ConceptNet dissimilarity within target-distractor sets.

Label for Target	Label for Distractor	THINGS Dissimilarity	ConceptNet Dissimilarity	Target Picture	Distractor Picture
baby cow	sheep	0.614	-1.493		
baby cow	camel	-0.146	-0.313		
baby cow	piglet	0.003	-0.249		
baby cow	pug	0.065	0.776		
baby cow	turkey	0.018	0.816		
baby cow	porcupine	0.081	1.193		
breakfast	quesadilla	-0.491	-0.639		
breakfast	brownie	-0.767	-0.353		
breakfast	crepe	-0.431	0.002		
breakfast	sushi	0.197	0.131		
breakfast	chips	-0.272	0.406		
breakfast	bean	1.451	0.722		
calzone	croissant	-1.170	-1.386		
calzone	enchilada	-1.064	-1.098		
calzone	sauerkraut	0.612	-0.460		
calzone	seafood	1.365	-0.319		
calzone	caramel	1.481	0.151		
calzone	berry	1.078	1.181		
casserole	egg roll	-1.553	-1.670		
casserole	vegetable	0.841	-1.469		
casserole	burrito	-1.340	-1.074		
casserole	guacamole	1.037	-1.068		
casserole	stuffing	-1.752	-0.915		
casserole	pickle	0.932	-0.295		
cheetah	walrus	0.140	-0.023		
cheetah	dalmatian	-0.430	0.377		
cheetah	rat	-1.113	0.445		
cheetah	pigeon	1.367	0.514		
cheetah	rooster	-0.442	0.893		
cheetah	beaver	-0.980	1.059		
cougar	hedgehog	-0.287	1.074		
cougar	gopher	-0.034	-0.498		
cougar	toucan	0.588	0.785		
cougar	platypus	1.223	0.754		
cougar	manatee	1.480	0.258		
cougar	snake	1.613	0.314		
deer	moose	0.199	-3.908		
deer	reindeer	-0.650	-1.873		
deer	raccoon	-0.159	-1.176		
deer	hyena	-0.740	-0.290		
deer	hawk	1.317	0.371		
deer	mongoose	-0.346	0.706		
dip	macaroni	0.455	0.262		
dip	fudge	-0.561	0.286		
dip	scrambled egg	-1.127	0.433		
dip	cashew	0.648	0.492		
dip	mousse	0.273	0.521		
dip	pistachio	1.396	0.899		
dog	horse	-0.046	-0.538		
dog	pig	0.076	-0.359		
dog	donkey	-0.778	-0.208		
dog	alligator	1.099	0.216		
dog	tiger	-1.363	0.301		
dog	tick	1.444	0.440		
duckling	pelican	1.641	-0.597		
duckling	squirrel	-0.877	-0.174		
duckling	hippopotamus	-0.594	0.085		
duckling	chipmunk	-0.824	0.241		
duckling	polar bear	1.472	0.514		
duckling	scorpion	1.568	1.728		
fast food	stir fry	-1.185	-0.255		
fast food	gravy	1.228	0.189		
fast food	bread	-0.056	0.335		
fast food	piecrust	0.773	0.493		
fast food	sorbet	1.591	0.761		
fast food	banana	-0.020	0.856		
frog	bee	-0.117	-0.654		
frog	dragonfly	0.931	-0.650		
frog	butterfly	1.012	-0.554		
frog	bug	1.357	0.180		
frog	cow	1.584	0.201		
frog	mosquito	1.294	0.316		
gazelle	antelope	-1.407	-2.672		
gazelle	aardvark	-1.544	-0.134		
gazelle	wolf	-0.761	-0.038		
gazelle	rabbit	-1.437	0.191		
gazelle	koala	-1.431	0.572		
gazelle	groundhog	-1.167	1.187		
gorilla	meerkat	-0.518	-1.037		
gorilla	otter	-0.376	-0.108		
gorilla	weasel	-0.498	0.039		
gorilla	chinchilla	-1.082	0.565		
gorilla	rattlesnake	1.511	0.893		
gorilla	chihuahua	-0.347	0.964		
guinea pig	mouse	-1.130	-0.674		
guinea pig	boar	-0.959	0.033		
guinea pig	lizard	0.418	0.260		
guinea pig	goose	-0.560	1.097		
guinea pig	pheasant	0.350	1.230		
guinea pig	vulture	1.442	1.435		
hamster	monkey	-0.988	-1.097		
hamster	panda	-0.500	-0.459		
hamster	rhinoceros	-1.244	0.497		
hamster	warthog	-1.094	0.578		
hamster	seal	0.696	1.347		
hamster	crow	1.511	1.137		
jalapeno	zucchini	-1.368	-1.634		
jalapeno	radish	-0.386	-1.473		
jalapeno	eggplant	1.395	-1.388		
jalapeno	cantaloupe	0.428	-0.847		
jalapeno	raspberry	1.328	0.027		
jalapeno	fig	1.169	0.807		
leopard	panther	-1.594	-4.123		
leopard	coyote	-1.342	0.016		
leopard	goat	-0.350	0.201		
leopard	badger	-1.565	0.264		
leopard	kangaroo	-1.173	0.311		
leopard	puffin	0.268	1.100		
llama	lamb	0.385	-0.196		
llama	bison	-1.115	-0.006		
llama	skunk	-0.624	0.233		
llama	toad	-0.304	0.553		
llama	fox	-0.940	0.647		
llama	flamingo	1.070	0.731		
orangutan	giraffe	-0.467	-1.292		
orangutan	cockatoo	1.205	-0.548		
orangutan	sloth	-1.293	0.123		
orangutan	owl	1.386	0.216		
orangutan	cat	-0.993	0.324		
orangutan	alpaca	0.219	0.653		
pony	ferret	1.545	-0.238		
pony	lion	-1.177	0.368		
pony	chick	-0.415	0.575		
pony	bear	-1.240	0.822		
pony	duck	1.359	1.159		
pony	bat	-0.534	1.300		
quiche	lasagna	-1.134	-3.412		
quiche	french fries	-0.639	-0.643		
quiche	spareribs	0.657	-0.577		
quiche	cracker	0.300	-0.318		
quiche	peanut	1.230	0.306		
quiche	gumdrop	1.453	0.681		
scallop	mussel	1.301	-2.916		
scallop	calamari	-0.772	-1.605		
scallop	cordon bleu	-1.156	-0.841		
scallop	cornbread	-0.947	-0.520		
scallop	ready meal	-0.604	0.751		
scallop	muffin	-0.337	0.751		
wrap	cucumber	1.120	0.550		
wrap	wafer	-0.641	0.863		
wrap	soup	0.555	0.933		
wrap	fruitcake	-1.085	1.031		
wrap	bell pepper	1.169	1.037		
wrap	eclair	-0.058	1.314		
